# Soil Microbial Biomass, Basal Respiration and Enzyme Activity of Main Forest Types in the Qinling Mountains

**DOI:** 10.1371/journal.pone.0067353

**Published:** 2013-06-28

**Authors:** Fei Cheng, Xiaobang Peng, Peng Zhao, Jie Yuan, Chonggao Zhong, Yalong Cheng, Cui Cui, Shuoxin Zhang

**Affiliations:** 1 College of Forestry, Northwest A&F University, Yangling, Shaanxi, China; 2 Qinling National Forest Ecosystem Research Station, Northwest A&F University, Yangling, Shaanxi, China; Universidad de Salamanca, Spain

## Abstract

Different forest types exert essential impacts on soil physical-chemical characteristics by dominant tree species producing diverse litters and root exudates, thereby further regulating size and activity of soil microbial communities. However, the study accuracy is usually restricted by differences in climate, soil type and forest age. Our objective is to precisely quantify soil microbial biomass, basal respiration and enzyme activity of five natural secondary forest (NSF) types with the same stand age and soil type in a small climate region and to evaluate relationship between soil microbial and physical-chemical characters. We determined soil physical-chemical indices and used the chloroform fumigation-extraction method, alkali absorption method and titration or colorimetry to obtain the microbial data. Our results showed that soil physical-chemical characters remarkably differed among the NSFs. Microbial biomass carbon (Cmic) was the highest in wilson spruce soils, while microbial biomass nitrogen (Nmic) was the highest in sharptooth oak soils. Moreover, the highest basal respiration was found in the spruce soils, but mixed, Chinese pine and spruce stands exhibited a higher soil *q*CO_2_. The spruce soils had the highest Cmic/Nmic ratio, the greatest Nmic/TN and Cmic/Corg ratios were found in the oak soils. Additionally, the spruce soils had the maximum invertase activity and the minimum urease and catalase activities, but the maximum urease and catalase activities were found in the mixed stand. The Pearson correlation and principle component analyses revealed that the soils of spruce and oak stands obviously discriminated from other NSFs, whereas the others were similar. This suggested that the forest types affected soil microbial properties significantly due to differences in soil physical-chemical features.

## Introduction

Soil microbes are the living component of soil organic matter [1]. Despite comprising only a small percentage of the total mass of soil organic matter, soil microbes are considered to exert essential influences on rate at which nutrient cycle through soil ecosystems, act as both soil available nutrition sources by mineralizing and sinks by immobilizing [2], [3], [4]. Owing to being central to ecosystem function, soil microbial indices have been included in soil quality monitoring programs.

Forest ecosystem is one of the essential terrestrial ecosystems [5]. Soil microorganisms involve in material cycles and energy transformation of forest ecosystems [6], [7], [8], in turn, the soil microbial activity can be controlled by forest types [9]. Different forest types which are composed of specific tree species are considered to have species-specific effects on soil properties by litters and root exudates. Therefore, it is likely that differences in tree species attributes will create distinctive soil environments [10]. Previous studies have demonstrated that size and activity of the soil microbial communities are commonly sensitive to the variation in soil physical and chemical properties [11], [12], [13], [14]. Consequently, the dominant tree species of forest types are capable of exerting important influences on the structure, function and activity of soil microbial communities via the quality and quantity of litters which input to soil, root architecture, nutrient requirements and decomposition process [10].

Currently, although there are a large number of publications about soil microbial communities in forest ecosystems, some of them refer to the size and activity of soil microbial communities, soil microbial-relating studies focusing on forest types are still limited, largely originating from broad research purposes. Furthermore, many studies are based on a large ecological scale at which it is difficult to overcome the impacts from climate (e.g., precipitation and temperature) and soil types [11], [15], [16], [17]. Additionally, forest age should not be ignored when quantifying indices of soil microbial communities [11], [17], [18]. Therefore, for concentrating on the relationship between forest type and microbial community and depicting accurately the pattern in characteristics of soil microbial community among forest types, investigating under a relatively uniform natural condition is definitely indispensible. Nevertheless, we should make clear that trying to realize this uniformity, it might be more appropriate that the studies are conducted at a small scale.

The Qinling Mountains are an important climate boundary between subtropical and warm temperate zones in China, where the typical vegetation of both climate zones assembles together with an astonishingly high biodiversity, is a veritable plant “kingdom” [19]. In 60s∼70s of the 20th century, most parts of Huoditang forest region in the Qinling Mountains experienced intensively selective logging, undoubtedly contributing to regeneration of diverse natural secondary forests (NSFs) that have the same stand age [20]. In these NSFs, the stands dominated by a single tree species are often adjacent to the stands dominated by another tree species, the mixed forests usually constitute between the two adjacent stands. Owing to close proximity to one-another, the NSF stands have little variability in the climate indices. Furthermore, loam soil is widespread under the most of forest stands in this region. As a result, the Houditang forest region provides us a favourable study area to find out linkages between soil microbes and forest types.

Previous studies recorded the differences in nutrition cycles and soil properties of these NSFs [21], [22], [23], yet soil microbial-associating records in the Qinling Mountains remains unknown. Over the past several decades, a variety of anthropogenic or natural disturbances, including loggings, fires, insect outbreaks, land-use change and global change continually happened, have been stimulating these NSFs to maintain dynamic changes. Therefore, a better understanding of the differences in soil environments and microbial community activity and the relationships between them in these NSFs may be a potential assistance for forecasting ecosystem attributes and processes. Thus, we selected five neighbouring stands of primary NSF types at the Huoditang forest region as study objects and hypothesized that the selected stands would have forest type-specific impacts on microbial properties by soil physical-chemical characters. Specifically, we hypothesized that the soil microbial biomass, basal respiration and soil enzyme activity would differ among the tested NSFs, and correlations between soil microbial and soil environmental factors would be observed.

## Materials and Methods

### Study Sites

The study was conducted at the Huoditang forest region on the south-facing slope of the Qinling Mountains. The altitude of Huoditang forest region is 800∼2500 m, the geographic coordinates are N33°18^′^∼33°28^′^ in latitude and E108°21^′^∼108°39^′^ in longitude. The annual average temperature is 8∼10°C, the annual precipitation is 900∼1200 mm, the frost-free period is 170 days, the climate belongs to the warm temperate zone. An abrupt and broken topography mainly consists of granite and gneiss. The mean slope is 35° and the mean soil depth is 45 cm. The soil is classified as loam. Armand pine (*Pinus armandi*), wilson spruce (*Picea wilsonii*), Chinese pine (*Pinus tabulaeformis*) and sharptooth oak (*Quercus aliena* var. *acuteserrata*) are the common tree species which distribute throughout most parts of the Qinling Mountains.

### Ethics Statement for Field Study

The Huoditang forest region is governed by the Huoditang Experimental Tree Farm which is an affiliate of Northwest A&F University. Normally, university stuff can be conducted field studies in this place without permissions from the authority. In present study, there were no required specific permissions and endangered or protected species to be included in this field investigation.

### Soil Sampling and Pretreatment

In September 2011, we selected soil samples from neighbouring stands of five main NSF types at the Qinling National Forest Ecosystem Research Station on the tree farm to reduce differences in microclimate caused by stand distance and to figure out effect of forest types on soil microbial communities. Four stands of these NSF types are dominated by Armand pine, wilson spruce, Chinese pine and sharptooth oak, respectively. One mixed stand is composed of Chinese pine and sharptooth oak. The stands of Chinese pine and sharptooth oak cluster together with the mixed stand, while the stands of other two NSFs are adjacent each other. At the Huoditang forest region, these stands share similar forest age and soil type and have a minor variation in precipitation and temperature. The environmental characteristics of these stands are shown in Table 1. For each NSF stand, three 10 m×10 m plots were established at intervals of 20 m. For an explicit research purpose, we did not sample the deeper soils, because of most of microbial biomass intensively distributing in the surface layer [24], [25], [26], [27], [28]. All soil samples were taken from the surface mineral layer (0∼10 cm) after litter removal. In each plot, five soil cores were collected using a soil corer (3 cm in diameter), and pooled into one composite sample. The soil samples were placed in plastic bags and taken to the laboratory using a cooling box. They were sieved (2 mm mesh) and stored at 4°C. Half of each sample was air-dried at room temperature for 2 weeks for analysis of soil physical-chemical parameters. In order to eliminate influences of soil water and temperature on microbes, another half of soil sample was adjusted to 60% of water holding capacity and placed in a sealed plastic container with 1 M NaOH for incubation at 25°C for 20 days. The NaOH was used to absorb evoluted CO_2_ during incubation.

**Table 1:** Environmental characteristics of five natural secondary forest types.

	Natural secondary forest type				
Environmental factor	Chinese pine	Sharptooth oak	Chinese pine+Sharptooth oak	Armand pine	Wilson spruce
Longitude	108°26′51″E	108°26′24″E	108°26′22″E	108°28′46″E	108°28′42″E
Latitude	33°26′10″N	33°26′10″N	33°26′02″N	33°27′17″N	33°27′58″N
Altitude (m)	1585	1554	1512	1963	2040
Slope degree (°)	35	38	36	15	5
Slope aspect	SW	SW	SW	S	E
DBH (cm)	16.12	12.77	14.28	18.57	19.14
Tree height (m)	15.85	9.61	12.35	13.56	12.23

DBH, diameter at breast height.

### Soil Physical-chemical Analysis

Physical-chemical parameters of the samples, including texture, particle size (PS), bulk density (BD), porosity, water holding capacity (WHC), pH, soil organic carbon (SOC), C/N ratio (C/N), total nitrogen (TN), total phosphorus (TP), total potassium (TK), ammoniacal nitrogen (NH_4_^+^-N), nitrate nitrogen (NO_3_^–^N), available phosphorus (AP), available potassium (AK) and nutrient elements (Ca, Mg and Na), were analyzed following the methods described by Liu et al. [29].

### Soil Microbial Biomass C and N

Microbial biomass carbon (Cmic) and microbial biomass nitrogen (Nmic) were estimated using the chloroform fumigation-extraction method [30]. Briefly, for each soil, three of six fresh subsamples (10 g dry weight equivalent) were fumigated with free-ethanol chloroform for 24 h in vacuum desiccators, another three were not fumigated as the control. The soil samples were extracted with 0.5 M K_2_SO_4_ (the ratio of soil/extractant was 1∶4) for 30 minutes (300 rpm) in an oscillator. The unfumigated soil samples were also subjected to a similar extraction. The resulting extracts were filtered. Cmic in filtrates was determined by potassium dichromate method [30], while Nmic was estimated by Kjeldahl method [31]. Cmic and Nmic were computed by differences between fumigated and unfumigated samples with a conversion factor of 0.38 for Cmic [30] and 0.54 for Nmic [31].

### Soil Basal Respiration

Alkali absorption method was performed for quantifying CO_2_ evolution. Briefly, moist soils (50 g dry weight equivalent) were adjusted to 60% of field holding capacity and pre-incubated at 25°C for 20 days. The incubated soils were spread on the bottom of 500-ml glass jars in which an absorption bottle with 10 ml NaOH (0.1 M) solution was hanged. After incubation at 25°C for 24 h, 2 ml of 0.5 M BaCl_2_ and 2 drops of phenolphthalein indicator were added into the bottles, and then titrated with 0.1 M HCl. The jars without soil served as the controls. The difference of consumed volume of HCl between the treatment and the control in titration was used to calculate the quantity of CO_2_ evolution from soil microbes, 1 ml 0.1 M consumed NaOH was equivalent to 2.2 mg CO_2_ [32]. The metabolic quotient (*q*CO_2_) was calculated as the ratio of respiration (µg CO_2_-C g^−1^ h^−1^) to Cmic.

### Soil Enzyme Assays

The activity of invertase (EC 3.2.1.26) was measured as described by Yao et al. [33], sucrose was used as a substrate. Briefly, 5 g of soil, 15 ml of 8% sucrose, 5 ml of phosphate buffer (pH = 5.5) and 5 drops of methylbenzene were mixed and incubated at 37°C for 24 h. After filtered, 1 ml of filtrate reacted with 3 ml of 3,5-dinitrosalicylic acid (DNS) in a 50-ml volumetric flask, followed by incubation in boiling bath for 5 min. The incubated solution was diluted to 50 ml. The absorbance was determined at 508 nm using a spectrophotometer. The invertase activity was expressed as mg glucose g^−1^ h^−1^ at 37°C.

The activity of urease (EC 3.5.1.5) was measured as described by Yao et al. [33], urea was used as a substrate. Briefly, 5 g of soil and 1 ml of methylbenzene was added in a 150-ml conical flask. After 15 min, 10 ml of 10% urea and 20 ml of citrate buffer (pH = 6.7) were mixed with the soil sample. The mixture was incubated at 37°C for 24 h. Following filtration, 1 ml of filtrate was transferred to a 50-ml volumetric flask, and mixed with 4 ml of sodium phenoxide and 3 ml of sodium hypochlorite. After diluted to 50 ml, the absorbance was determined at 578 nm using a spectrophotometer. The urease activity was expressed as µg NH_3_-N g^−1^ h^−1^ at 37°C.

The activity of acid phosphatase (EC3.1.3.2) was measured as described by Schneider et al. [34], *p*-nitrophenyl phosphate (*p*-NPP) was used as a substrate. Briefly, 5 g of soil and 20 ml of acetate buffer (pH = 5.2) and 1 ml of 100 mM *p*-NPP were added in a 150-ml conical flask. The reaction mixture was mixed and incubated at 30°C for 30 min. After incubation, 1 ml of 2 M CaCl_2_ and 4 ml of 0.2 M NaOH were added to terminate the reaction, followed by dilution and filtration. The absorbance of supernatants was determined at 405 nm using a spectrophotometer. The acid phosphatase activity was expressed as µg *p*-NP g^−1^ h^−1^ at 30°C.

The activity of catalase (EC 1.11.1.6) was measured as described by Li et al. [35], hydrogen peroxide was used as a substrate. Briefly, 5 g of soil, 40 ml of distilled water and 5 ml of 0.3% H_2_O_2_ were added in a 150-ml conical flask, followed by seal and oscillation at 120 rpm for 30 min. After addition of 5 ml of 1.5 M H_2_SO_4_ to terminate the reaction, the reaction mixture was filtered, 25 ml of filtrate was titrated with KMnO_4_. The catalase activity was evaluated with consumed volume of KMnO_4_ and expressed as ml (0.1 M KMnO_4_) g^−1^.

### Statistical Analysis

All samples were measured in triplicate. The selected variables were compared between the tested soils from the five NSF types using a one-way ANOVA with LSD’s post-hoc test. When equal variance was not assumed, Dunnett’s T3 post-hoc test was used. Significant differences were detected at the 0.05 level. Correlations between all measurable variables were estimated using Pearson’s *r* with *p*<0.05 significance threshold. The correlation matrix was also analyzed through principle component analysis but ratios. The SPSS 13.0 was run for data processing.

## Results

### Soil Properties of Five NSFs

Acquired data encompasses a wide range of soil physical-chemical condition of NSFs (Table 2 and 3). Sharptooth oak soils had more root biomass. These NSF soils tended to have higher silt than sand and clay. Silt contents in soils of Armand pine, wilson spruce and mixed stands were higher than those of Chinese pine and sharptooth oak stands. The oak soils had the lowest bulk density and highest soil porosity. In the stands of wilson spruce and Armand pine, soil water holding capacity was lower than that in other three NSFs. All soils were acidic, ranging from pH 3.95 to pH 6.06, except for soils of mixed stand which were markedly alkaline. Noticeably, the spruce soils was very distinguishing in soil chemical characteristics, since over half of the soil chemical variables (SOC, TN, TP, AP, Ca and Mg) were higher than those in other NSFs. The oak soils had the highest contents of TK, NH_4_^+^-N and AK. Additionally, the maximal soil C/N ratio was found in the Chinese pine soils, while the Armand pine and mixed stands were superior in the contents of NO_3_^–^N and Na.

**Table 2:** Soil physical factors of five natural secondary forest types.

	Natural secondary forest type				
Chemical factor	Chinese pine	Sharptooth oak	Chinese pine+Sharptooth oak	Armand pine	Wilson spruce
Root content	++	+++	++	++	++
Sand (%)	19.70(0.77)a	32.96(1.40)b	18.80(1.56)a	14.61(0.36)c	28.84(0.38)d
Silt (%)	52.01(1.64)a	51.16(1.09)a	64.42(2.35)b	66.05(2.51)b	65.14(1.61)b
Clay (%)	28.29(1.82)a	15.88(1.77)b	16.78(2.06)bc	19.35(1.49)c	6.20(0.08)d
BD (g/cm^3^)	1.08(0.08)a	0.89(0.05)b	1.14(0.04)a	1.11(0.04)a	1.06(0.03)a
Porosity (%)	3.29(0.20)a	24.12(1.14)b	16.20(1.95)c	13.69(0.65)c	20.19(2.68)d
WHC (%)	22(1)a	20(2)a	21(1)a	17(1)b	16(1)b

BD, bulk density; WHC, water holding capacity. Values are means±SD and letters denote significant differences among forest types (*P* = 0.05).

**Table 3:** Soil chemical factors of five natural secondary forest types.

	Natural secondary forest type				
Chemical factor	Chinese pine	Sharptooth oak	Chinese pine+Sharptooth oak	Armand pine	Wilson spruce
pH (H_2_O)	6.06(0.04)a	3.95(0.23)b	11.04(0.09)c	5.93(0.19)a	5.86(0.25)a
SOC (g/kg)	37.08(2.17)a	26.44(1.12)b	24.30(1.15)b	18.09(1.14)c	48.89(0.35)d
C/N	19.32(0.60)a	15.71(1.04)b	16.58(0.32)b	13.98(0.93)c	12.74(0.75)c
TN (g/kg)	1.92(0.17)a	1.69(0.04)abc	1.47(0.10)bc	1.30(0.17)bcd	3.85(0.20)e
TP (g/kg)	0.40(0.03)a	0.34(0.02)b	0.30(0.01)b	0.32(0.02)b	1.14(0.04)c
TK (g/kg)	23.34(1.25)a	28.31(0.67)b	20.65(1.01)c	19.29(1.12)c	15.18(2.51)d
NH_4_^+^-N (mg/kg)	14.01(0.25)a	20.87(0.18)b	12.45(0.28)c	18.86(0.06)d	20.10(0.28)e
NO_3_^–^N (mg/kg)	3.02(0.33)a	2.65(0.18)ab	3.10(0.09)acd	3.13(0.22)ad	2.57(0.22)b
AP (mg/kg)	4.51(0.14)a	2.50(0.17)b	3.17(0.08)b	2.96(0.20)b	26.11(1.20)c
AK (mg/kg)	214.80(5.67)a	248.15(10.59)b	160.25(7.99)c	164.15(7.49)c	215.35(8.24)a
Ca (g/kg)	4.22(0.22)a	1.72(0.29)b	3.42(0.22)c	2.20(0.31)b	11.47(0.32)d
Mg (g/kg)	11.90(1.74)a	8.39(1.23)b	11.05(1.85)ab	7.28(0.66)bc	16.33(1.84)d
Na (g/kg)	4.05(0.23)a	3.61(0.19)b	5.34(0.13)c	5.56(0.36)c	4.47(0.11)d

SOC, soil organic carbon; TN, total nitrogen; TP, total phosphorous; TK, total kalium; AP, available phosphorous; AK, available kalium. Values are means±SD and letters denote significant differences among forest types (P = 0.05).

SOC, soil organic carbon; TN, total nitrogen; TP, total phosphorous; TK, total kalium; AP, available phosphorous; AK, available kalium. Values are means±SD and letters denote significant differences among forest types (*P* = 0.05).

### Soil Microbial Biomass of five NSFs

**Figure 1:**
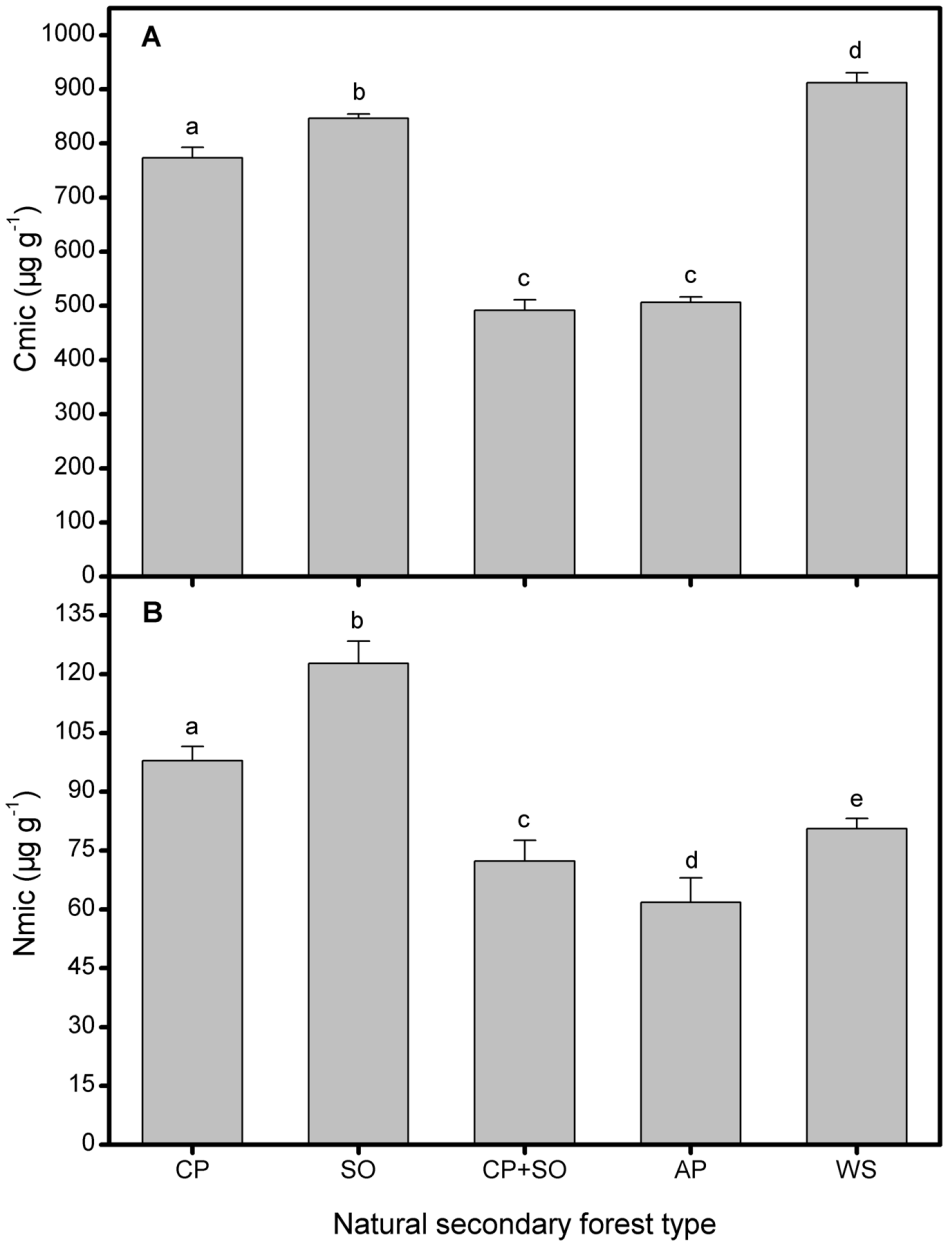
Soil microbial biomass of five natural secondary forest types. Soil (A) Cmic and (B) Nmic of each forest type. CP–Chinese pine, SO–Sharptooth oak, AP–Armand pine and WS–Wilson spruce. Values are means±SD and letters denote significant differences among forest types (*P* = 0.05).

The size of Cmic differed significantly among the NSF types of the Qinling Mountains, ranged from 491.93 to 912.29 µg g^−1^. Both the mixed and Armand pine stands had the lowest Cmic, while the spruce soils contained the highest Cmic concentration which was almost twice as high as the mixed and Armand stands (Fig. 1). Likewise, soil Nmic ranged from 61.79 to 122.79 µg g^−1^, also being affected by forest types, however, the highest and the lowest Nmic concentrations occurred in the oak and Armand pine soils, respectively (Fig. 1).

### Soil Basal Respiration and qCO_2_ of Five NSFs

**Figure 2:**
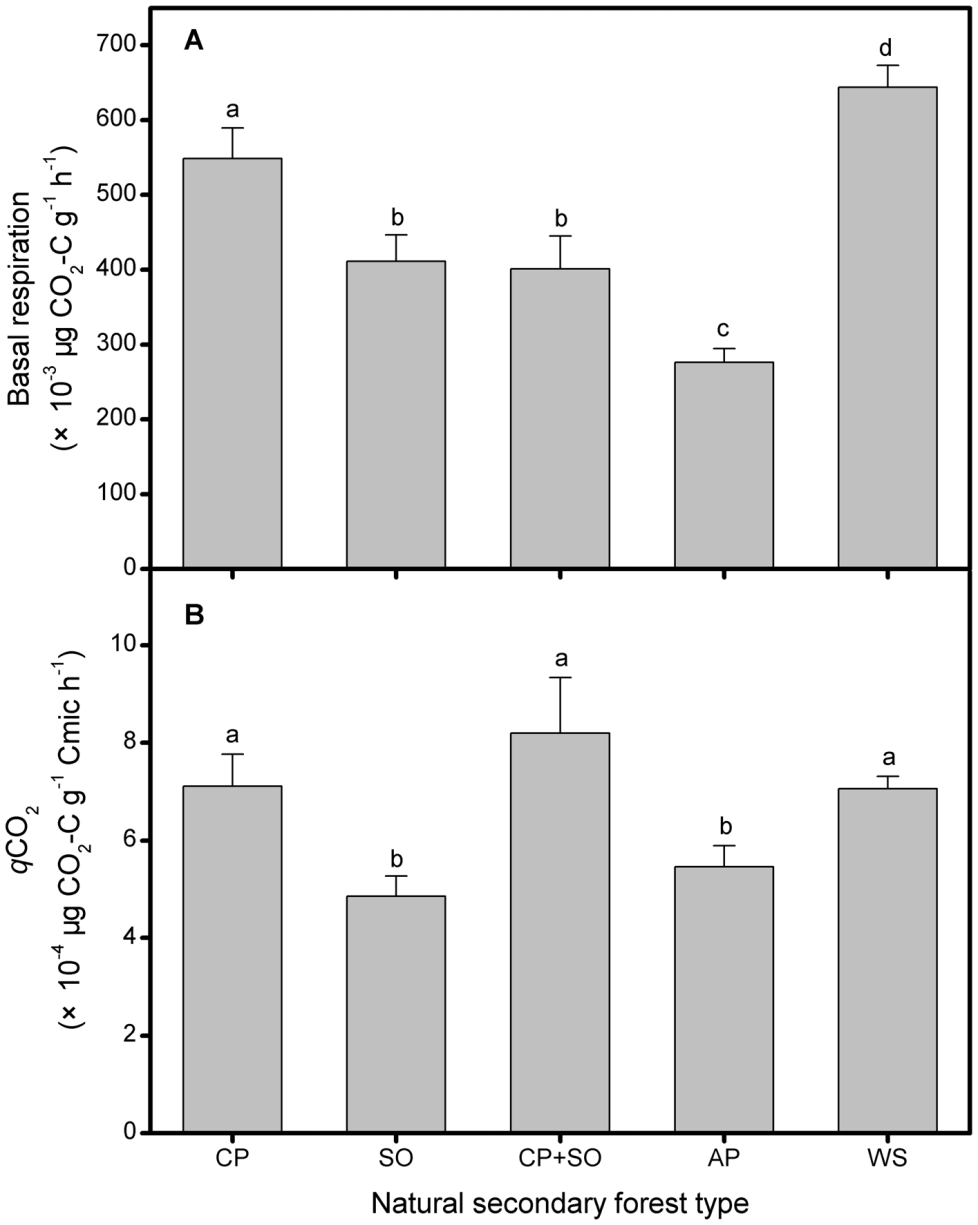
Soil basal respiration and metabolic quotient of five natural secondary forest types. Soil (A) basal respiration and (B) metabolic quotient of each forest type. CP–Chinese pine, SO–Sharptooth oak, AP–Armand pine and WS–Wilson spruce. Values are means±SD and letters denote significant differences among forest types (*P* = 0.05).

The pattern of soil basal respiration was strongly controlled by forest types as well as metabolic quotient (*q*CO_2_) (Fig. 2). The maximum basal respiration was found in the spruce soils, up to 644.33×10^−3^ µg CO_2_-C g^−1^ h^−1^, but in the Armand pine soils, the basal respiration drastically decreased to 276.44×10^−3^ µg CO_2_-C g^−1^ h^−1^. The basal respiration varied very little between the soils of oak forest and mixed forest. However, perhaps driven largely by differences in Cmic, the varying pattern of *q*CO_2_ did not follow basal respiration among the five NSFs (Fig. 2). The five NSFs were obviously divided into two groups by data analysis,the mixed, Chinese pine and spruce stands as a group exhibited a higher soil *q*CO_2_ than the other two forest types.

### Cmic/Nmic, Nmic/TN and Cmic/Corg Ratios of Five NSFs

**Figure 3:**
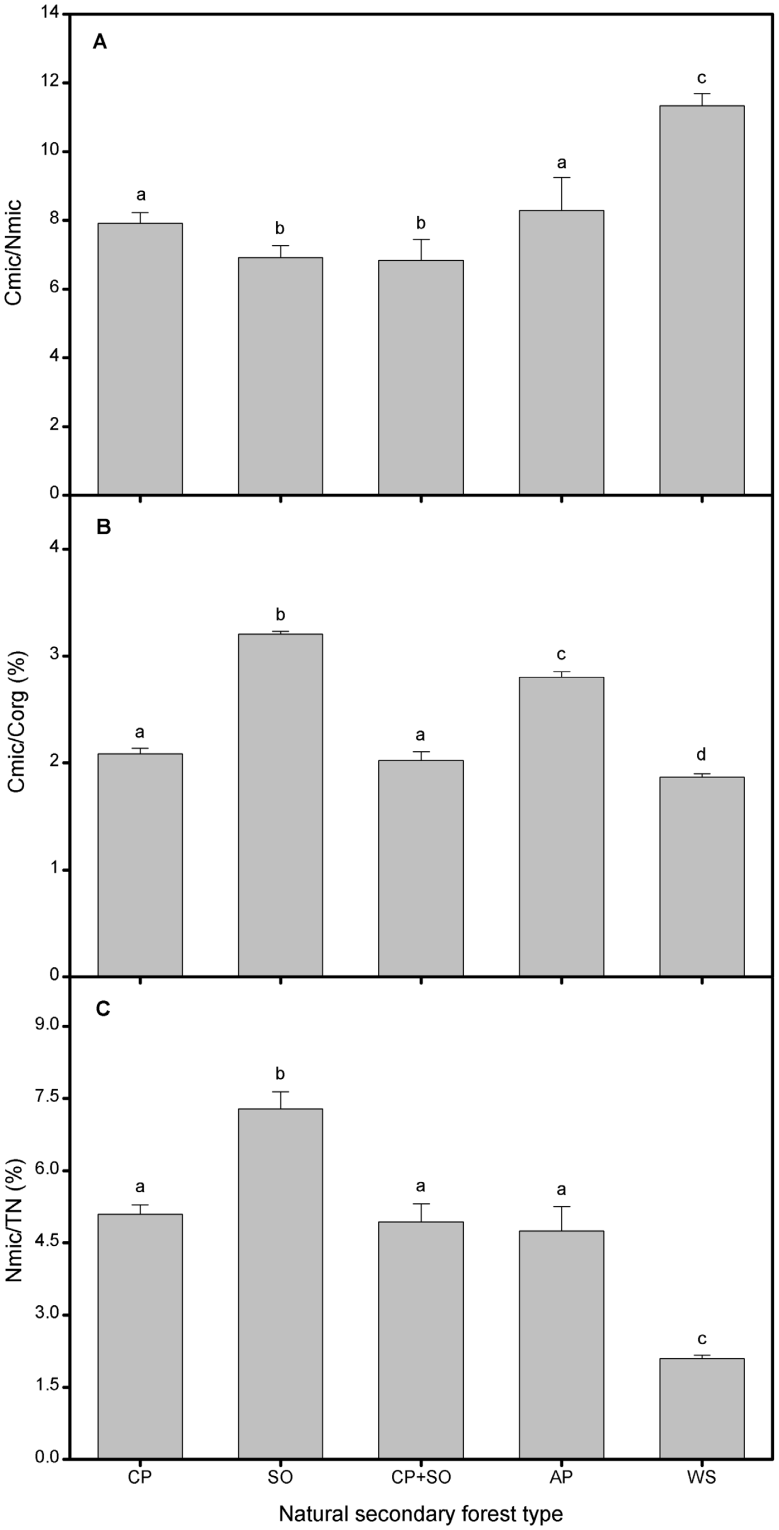
Biomass-relating ratios of five natural secondary forest types. Soil (A) Cmic/Nmic, (B) Cmic/Corg and (C) Nmic/TN of each forest type. CP–Chinese pine, SO–Sharptooth oak, AP–Armand pine and WS–Wilson spruce. Values are means±SD and letters denote significant differences among forest types (*P* = 0.05).

There were significant effects of forest types on microbial biomass-relating ratios (Fig. 3). In addition to Cmic/Nmic ratio, the ratios of Nmic/TN and Cmic/Corg in the spruce soils were lower than those in other NSFs, while the lowest Cmic/Nmic ratio appeared in the soils of oak and mixed stands. The highest ratios of Nmic/TN and Cmic/Corg were found in the oak soils.

### Enzyme Activities of Five NSFs

**Figure 4:**
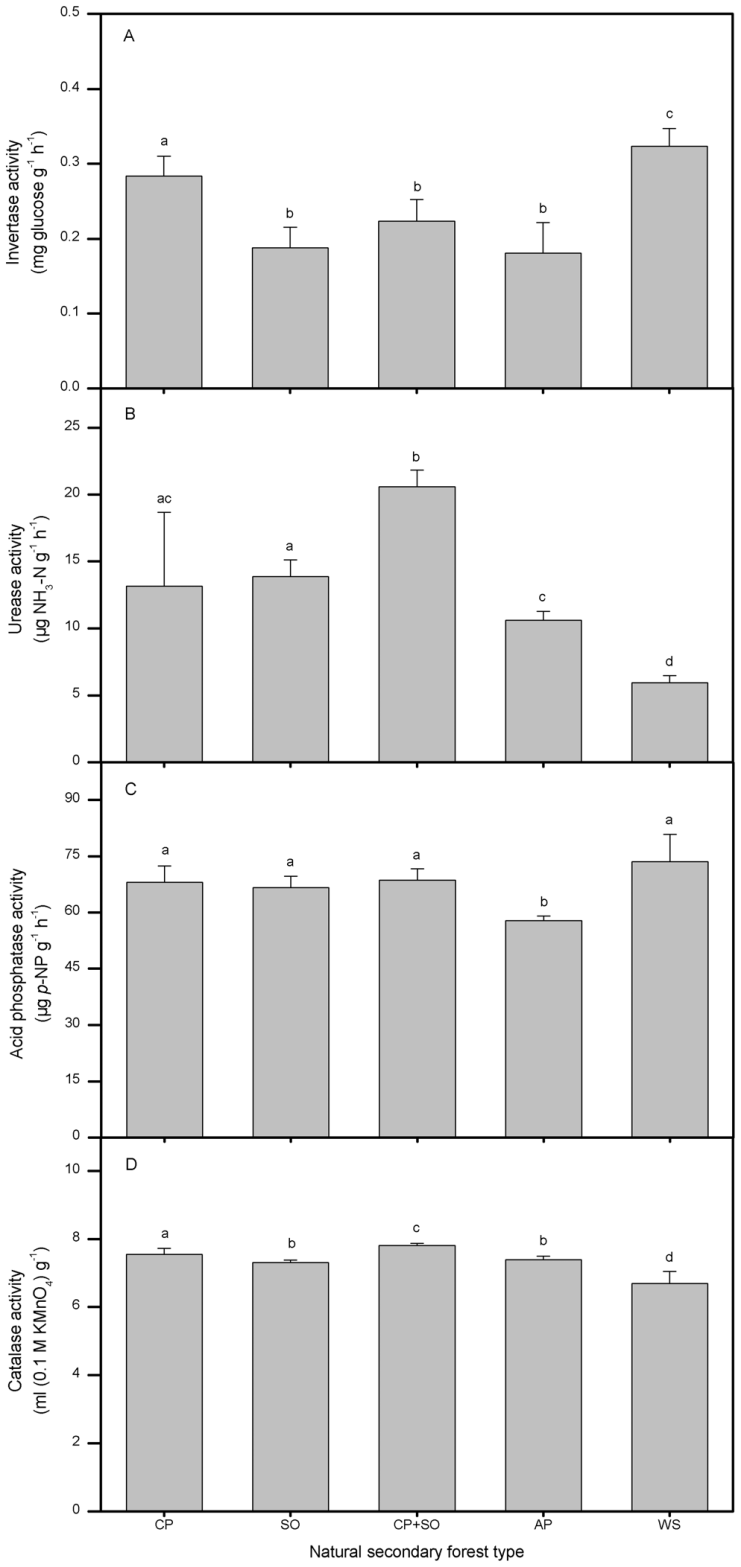
Soil enzyme activities of five natural secondary forest types. Soil (A) invertase, (B) urease, (C) acid phosphatase and (D) calatase activities of each forest type. CP–Chinese pine, SO–Sharptooth oak, AP–Armand pine and WS–Wilson spruce. Values are means±SD and letters denote significant differences among forest types (*P* = 0.05).

Enzyme activities also showed wide forest type-dependent variations, ranging from 57.91 to 73.63 µg *p*-NP g^−1^ h^−1^ for acid phosphatase, from 5.93 to 20.59 µg NH_3_-N g^−1^ h^−1^ for urease and from 0.18 to 0.32 mg glucose g^−1^ h^−1^ for invertase, respectively; catalase activity ranged from 6.70 to 7.81 ml (0.1 M KMnO_4_) g^−1^ (Fig. 4). Except for the lowest activity in soils of Armand pine, other NSFs did not differ significantly in acid phosphatase activity. The activities of both catalase and urease in wilson spruce stand were lower than those in other NSFs. On the contrary, these two enzymes in the soils of mixed stand had remarkably higher activity than in other soils. Invertase showed a higher activity in the soils of wilson spruce and Chinese pine stands.

### Multivariate Analysis of Soil Physical-chemical and Microbial Properties

Pearson correlation matrix revealed that a large number of soil variables were significantly correlated with each other (Table 4). Diverse correlations were found between distinct soil variables, but we did not attempt to interpret these since they were not the focus of this study. For a few physical variables, negative correlation was observed between soil pH and Cmic, and positive correlations between pH and activities of calatase and urease, the reminder of physical variables had a weak correlation with the biological variables. By contrast, closer correlations were found between chemical and biological variables. The activities of invertase and acid phosphatase were positively correlated with SOC, TN, TP, AP, Ca, Mg, Cmic and BR (basal respiration), while significantly positive correlations among pH, NO_3_^–^N and activities of catalase and urease were found. Additionally, Both Cmic and BR were positively correlated with SOC, TN, TP, AP, Ca and Mg,and negatively correlated with NO_3_^–^N. Nmic was positively related with sand, TK, AK and Cmic, negatively related with silt, BD, pH, NO_3_^–^N and Na.

**Table 4:** Correlation coefficients (*r*) between soil microbial and physical-chemical factors.

Soil factor	Sand	Silt	Clay	BD	WHC	Porosity	pH	SOC	TN	TP	TK	NH_4_^+^-N	NO_3_^–^N	AP	AK	Ca	Mg	Na	Invertase	ACP	Catalase	Urease	Cmic	BR
Silt	−0.443																							
Clay	−**0.540**	−**0.516**																						
BD	−**0.845**	**0.682**	0.167																					
WHC	−0.115	−**0.661**	**0.730**	−0.046																				
Porosity	**0.689**	0.159	−**0.808**	−**0.566**	−0.454																			
pH	−**0.520**	0.498	0.029	**0.736**	0.299	−0.184																		
SOC	0.473	−0.065	−0.390	−0.059	−0.219	−0.030	−0.198																	
TN	0.512	0.217	−**0.693**	−0.060	−**0.547**	0.255	−0.206	**0.922**																
TP	0.409	0.338	−**0.708**	0.041	−**0.630**	0.230	−0.167	**0.882**	**0.990**															
TK	0.318	−**0.853**	0.497	−**0.687**	**0.672**	0.068	−0.328	−0.418	−**0.593**	−**0.694**														
NH_4_^+^−N	**0.607**	−0.026	−**0.555**	−**0.665**	−**0.701**	**0.651**	−**0.807**	0.141	0.372	0.368	0.022													
NO_3_^–^N	−**0.943**	0.231	**0.684**	**0.701**	0.383	−**0.654**	**0.564**	−**0.650**	−**0.741**	−**0.669**	−0.019	−**0.706**												
AP	0.401	0.384	−**0.743**	0.056	−**0.658**	0.266	−0.139	**0.856**	**0.984**	**0.998**	−**0.715**	0.375	−**0.662**											
AK	**0.864**	−**0.762**	−0.110	−**0.877**	0.098	0.290	−**0.753**	0.478	0.367	0.253	0.510	**0.533**	−**0.795**	0.218										
Ca	0.313	0.371	−**0.648**	0.173	−**0.541**	0.124	−0.021	**0.898**	**0.973**	**0.985**	−**0.742**	0.203	−**0.569**	**0.983**	0.158									
Mg	0.308	0.198	−0.481	0.203	−0.228	−0.015	0.139	**0.936**	**0.901**	**0.884**	−**0.605**	−0.064	−0.495	**0.873**	0.190	**0.941**								
Na	−**0.789**	**0.866**	−0.059	**0.816**	−0.316	−0.142	**0.640**	−0.454	−0.265	−0.138	−**0.601**	−0.330	**0.676**	−0.097	−**0.973**	−0.073	−0.180							
Invertase	0.185	0.066	−0.240	0.240	−0.128	−0.263	0.042	**0.934**	**0.821**	**0.805**	−**0.543**	−0.129	−0.377	**0.780**	0.216	**0.868**	**0.943**	−0.232						
ACP	**0.558**	−0.109	−0.431	−0.105	0.074	0.172	0.140	**0.808**	**0.725**	**0.654**	−0.204	−0.094	−**0.595**	**0.642**	0.406	**0.701**	**0.848**	−0.437	**0.734**					
Catalase	−**0.567**	−0.179	**0.709**	0.308	**0.774**	−0.421	**0.570**	−**0.694**	−**0.864**	−**0.869**	0.454	−**0.759**	**0.802**	−**0.867**	−0.467	−**0.774**	−**0.570**	0.289	−0.513	−0.350				
Urease	−0.275	−0.165	0.417	0.162	**0.770**	−0.105	**0.672**	−**0.575**	−**0.700**	−**0.733**	0.471	−**0.712**	**0.547**	−**0.721**	−0.347	−**0.640**	−0.404	0.180	−0.457	−0.095	**0.901**			
Cmic	**0.833**	−0.492	−0.334	−**0.652**	−0.151	0.255	−**0.667**	**0.786**	**0.716**	**0.628**	0.109	**0.536**	−**0.892**	**0.595**	**0.907**	**0.554**	**0.552**	−**0.849**	**0.557**	**0.611**	−**0.727**	−**0.590**		
BR	0.478	−0.165	−0.302	−0.070	−0.055	−0.078	−0.128	**0.982**	**0.855**	**0.798**	−0.315	0.022	−**0.609**	**0.768**	0.499	**0.830**	**0.925**	−0.511	**0.934**	**0.848**	−**0.577**	−0.440	**0.773**	
Nmic	**0.756**	−**0.885**	0.109	−**0.869**	0.416	0.241	−**0.560**	0.194	0.029	−0.100	**0.768**	0.294	−**0.567**	−0.133	**0.897**	−0.170	−0.054	−**0.934**	−0.021	0.274	−0.105	0.042	**0.674**	0.266

In bold, significant values at the 0.05 level. BD, bulk density; WHC, water holding capacity; SOC, soil organic carbon; TN, total nitrogen; TP, total phosphorous; TK, total kalium; AP, available phosphorous; AK, available kalium; ACP, acid phosphatase; Cmic, microbial biomass carbon; BR, basal respiration; Nmic, microbial biomass nitrogen.

**Figure 5:**
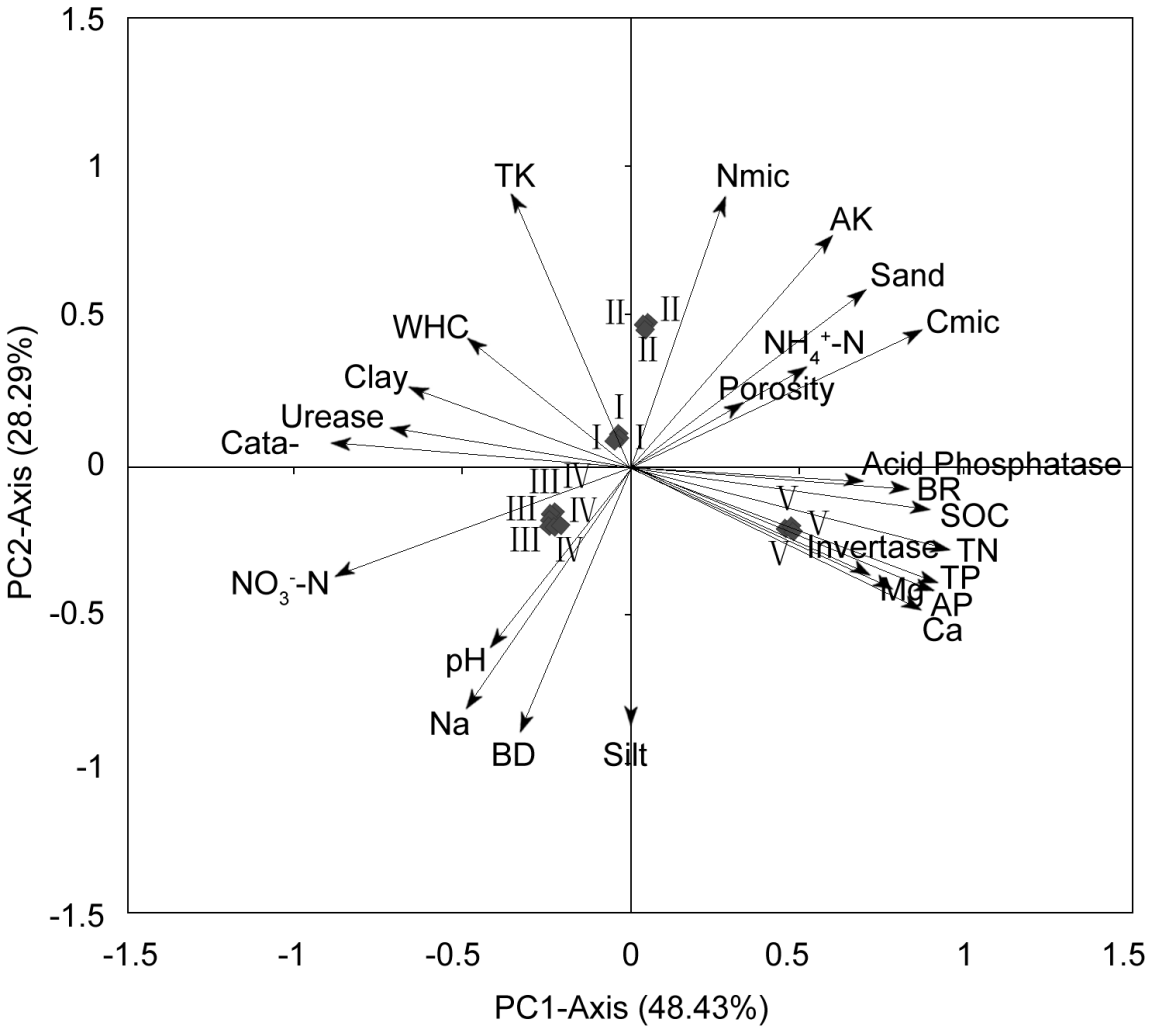
PCA-ordination biplot (PC1 vs PC2) of soil samples from five forest types and 25 soil microbial and physico-chemical factors. BD–bulk density, WHC–water holding capacity, SOC–soil organic carbon, TN–total nitrogen, TP–total phosphorous, TK–total kalium, AP–available phosphorous, AK–available kalium, Cmic–microbial biomass carbon, BR–basal respiration, Nmic–microibal biomass nitrogen. I–Chinese pine, II–Sharptooth oak, III–Chinese pine+Sharptooth oak, IV–Armand pine, V–Wilson spruce.

Through a principal component analysis, we retained four components. The first two components (PC1 and PC2) explained 48.43% and 28.29% of the total variance, respectively, while 15.40% and 7.29% of the total variance were explained by other two (PC3 and PC4), respectively. The PC1 and PC2 were chosen to draw a biplot (Fig. 5), as a result of explaining 76.73% of the total variance together. Note that the PC1 was mainly weighted by chemical (SOC, TN, TP, AK, Ca, Mg and NO_3_^–^N) and biological (enzymatic activities, Cmic and BR) variables together with sand and clay contents. In the tested soil samples, coordination changes happened in these variables, sand, SOC, TN, TP, AK, Ca, Mg, invertase, acid phosphatase, Cmic and basal respiration positively correlated to each other, and negatively correlated with clay, NO_3_^–^N and activities of calatase and urease. In comparison with PC1, the PC2 was weighted by only a few of variables, physical variables (Silt, BD and pH) exhibited a more powerful influence on discrimination in soil samples. Sand, TK, AK and Nmic were positively correlated to each other and negatively correlated with silt, BD, pH and Na. Besides, the selected soils were classified in three groups. Specifically, the soils of spruce forest and oak forest clustered as one group, respectively, being separated from other soils which were clustered in a broad area.

## Discussion

Contents of certain elements (C, N, P and S) by the chloroform fumigation-extraction method are usually estimated to express as soil microbial biomass, especially, Cmic and Nmic [36], [37], [38], [39]. In our study, we interpreted differences in soil microbial biomass (Cmic and Nmic) among the NSF types to be the result of soil physical-chemical properties. It should be noted that positive correlation between Cmic and SOC frequently was observed, indicating size of soil organic C pools [1]. Both Cmic and Nmic were slightly higher than previous publication which investigated the soil microbial biomass of Chinese pine and sharptooth oak forests at the Huoditang forest region [40], the increase in the microbial biomass may be ascribed to one-decade accumulation of soil organic carbon, so it is not surprising that wilson spruce forest had the highest Cmic, given a high level of SOC. Nmic coordinatedly varied with Cmic among the NSFs, yet unlike Cmic, the maximum Nmic was found in the oak forest other than the spruce forest. Unexpectedly, although the N restriction occurs in a variety of ecosystems [41], in our study lacking explicit associations of Nmic and other soil factors drives us to speculate that N availability was not a primary limiting factor to soil microbial growth at the Huoditang forest region, which was supported by a fact that none of Cmic/Nmic exceeded soil C/N in all tested soils [37], [42]. As for mineralized N, soil microbial communities in these soils were more inclined to absorbing NH_4_^+^-N than NO_3_^–^N since microbes consume energy for NO_3_^–^N absorption [43], [44]. Previous studies demonstrated that the fungal Cmic/Nmic ratio ranged from 4∼15, while this ratio for bacteria ranged from 3∼5 [45], revealing that this ratio have a potential to indicate the proportion of fungi and bacteria in soils, namely, the ratio increasing with fungal abundance [46]. In the coniferous forests, soil fungal populations were more predominated than bacterial populations, especially in wilson spruce forest [46], which resulted in the coniferous forests exceeding broadleaved and mixed forests in soil Cmic/Nmic. Cmic/Corg ratio reflects the contribution of microbial biomass to soil organic carbon [47]. Soil labile C availability may also be assessed by this ratio, in reverse, it can indicate the fraction of recalcitrant organic matter in the soil [48]. As a rule, Cmic is largely dependent on the soil labile C as C source, the high Cmic/Corg ratio is indicative of soil labile C accumulation and favourable environment for microbial growth, whereas the low ratio usually has a closely linkage to the poor quality of organic matter. The highest ratio of Cmic and Corg indicated the highest substrate availability in the soils of oak forest, while recalcitrant C compounds prevailed in the soil C pools under coniferous forests, presumably driving fungal domination in the coniferous forest soils [49]. It is worthwhile to note that in present study despite of low level of SOC under the oak forest, the rich Cmic contents might be attributed to the high substrate availability as suggested by the high Cmic/Corg. Nmic/TN ratio usually reflects the active N pool, relating to soil N availability and mineralization [50]. A high ratio represents a speed N transformation and supply ability [51]. In this study, the highest Nmic/TN ratio indicated that the oak forest had a higher potential in soil N mineralization, while the ability of soil N supply in the spruce stand was lower than that in other stands. To improve ecosystem stability and health in the spruce forest, further studies on soil N transformation and regulation are required. Moreover, the demand of soil microorganisms in mineral soils to P element was probably aggravated by a part of P being absorbed by plants in the soil organic layer as proved by the significantly positive correlations between Cmic and TP, AP [52]. Metal elements participate in microbial cell construction and metabolism. Indeed, in our study the spruce soils are rich in potassium, calcium and magnesium, which may trigger the microbial biomass. The demands of the cell growth to metal elements well explained the positive correlations of microbial biomass and potassium, calcium, magnesium [53]. Generally, the high sand content and low pH are disadvantageous to microbial growth [12], [54], [55], [56], [57]. On the contrary, the Pearson’s correlation indicated strongly positive correlation between microbial biomass and sand content and negative correlation between microbial biomass and pH. However, there is little evidence to explain these contradictory results to previous studies.

All the soil factors controlling Cmic had significant effects on soil basal respiration as described by the correlation analysis. The basal respiration gives an estimate of total microbial activity, reflecting both the quantity and quality of the carbon sources [58]. A higher soil respiration supported our notion that the spruce forest soils had a higher soil microbial activity, suggesting rapid decomposition of organic residues that makes nutrients available for the consequent stimulation of heterotrophic microorganisms in spruce stand [59]. In addition, it seems that the lower soil microbial metabolic quotient (*q*CO_2_) showed lower soil chemical stress to microorganisms, more fruitful C utilization efficiency, less energy demand in microbial biomass maintenance and better soil quality in the oak and Armand pine forests [49], [60], [61], [62].

The tested soil invertase, urease, and acid phosphatase in present study play an essential role in C, N, and P cycles in soils, respectively. Catalase activity is recognized to link with soil respiratory intensity and microbial activity, reflecting the soil microbial processes to some extent. Among the tested forest types, the varying trend of invertase activity was almost in accordance with SOC and TN, which reflects the laws in accumulation and decomposition of soil organic matter in the wilson spruce forest [63], [64], [65]. Acid phosphatase is directly responsible for phosphorus cycle in forest soils. Enzyme activity positively associated with TP and available P, suggesting that wilson forest was more effective in soil organic P-mineralizing [66], [67]. Probably, ammonium N in the tested soils was sufficient to maintain the demand of plants and microorganisms, resulting in no positive correlations between the urease activity and soil microbes, which is consistent with the negative feedback mechanism of soil N supply and urease activity, also indirectly verifying the view of N failing to restrict microbial growth. Besides, the soil urease activity may be affected by the soil microorganism quantity, SOC, TN and available P. The catalase activity is commonly recognized to relate with SOC, but our result shows a negative correlation of catalase activity and SOC content. Therefore, it forced us to speculate that other factors may potentially regulate catalase activity. For instance, Lorenz et al. [68] observed that tannin in the litter of coniferous tree species acts as an inhibitor for catalase activity, this inhibition effect of tannin on catalase activity was also reported in other literatures [69], [70]. On the whole, the soils from wilson spruce forest were higher in transformation of C and P than other NSFs, while the soils of oak forest had an advantage over N cycle and had less inhibitory effect of tannin on calatase.

The PCA allowed us to detect the subtle differences in soil physical-chemical and microbial factors among the studied forest types. Particularly, the spruce soils were apparently discriminated from the other NSFs due to half of chemical factors and a few physical and microbial factors clustering nearby the spruce soils. Combining with Ca and Mg, TP and AP which were closely associated with soil particularity of wilson spruce forest were likely to act as stimulating factors that positively affected the size of Cmic as well as acid phosphatase activity. Additionally, the high SOC and TN contents as material basis largely contribute to the high Cmic, basal respiration and enzymatic activities. However, this was not a guarantee for high C-use efficiency (*q*CO_2_), as a result of more recalcitrant C in the wilson spruce soils. By all accounts, the soil factors including microbial and physical-chemical variables mentioned above cause the striking discrimination of spruce soils from other soils to a great extent. In addition, the discrimination of oak forest soils mainly derives from integrated contribution of soil physical (Clay, WHC, Sand and Porosity), chemical (TK, AK and NH_4_^+^-N) and microbial (Cmic and Nmic) factors. Except for the factors mentioned above, the remainder exerted synthetically positive or negative influences on the other three forests. It is worth to note that the mixed forest of Chinese pine and sharptooth oak was similar to Armand pine forest in respect of effects of soil factors.

Intensive loggings happened in the mid 20th century had far-reaching influences on spatial pattern of original forests in the Qinling Mountains [20]. For example, the Chinese pines or/and Armand pines gradually occurred and prevailed in the stands dominated by oaks before loggings. But after a long-term recovery, nowadays, partial these stands have turned to pine-oak forests and have an apparent trend to re-success to climax communities dominated by oaks [71]. Nevertheless, intensity of such man-made interference is restricted by multiple factors, causing somewhat indeterminacy which presumably is responsible for degradation of some Chinese pine stands. Moreover, combining with current climate change, there is no doubt that this indeterminacy is strengthened, which will make how the forests success in the future more unpredictable. As a consequence, the present study will perhaps provide a potential insight with us into accurate predictions for impact of forest type transformation on both soil physical-chemical environment and microbial community.
